# What are the visuo-motor tendencies of omnidirectional scene free-viewing in virtual reality?

**DOI:** 10.1167/jov.22.4.12

**Published:** 2022-03-24

**Authors:** Erwan Joël David, Pierre Lebranchu, Matthieu Perreira Da Silva, Patrick Le Callet

**Affiliations:** 1 Department of Psychology, Goethe-Universität, Frankfurt, Germany; 2 LS2N UMR CNRS 6004, University of Nantes and Nantes University Hospital, Nantes, France; 3 LS2N UMR CNRS 6004, University of Nantes, Nantes, France

**Keywords:** virtual reality, gaze-contingent paradigm, artificial vision loss, eye-movements, time-dynamics

## Abstract

Central and peripheral vision during visual tasks have been extensively studied on two-dimensional screens, highlighting their perceptual and functional disparities. This study has two objectives: replicating on-screen gaze-contingent experiments removing central or peripheral field of view in virtual reality, and identifying visuo-motor biases specific to the exploration of 360 scenes with a wide field of view. Our results are useful for vision modelling, with applications in gaze position prediction (e.g., content compression and streaming). We ask how previous on-screen findings translate to conditions where observers can use their head to explore stimuli. We implemented a gaze-contingent paradigm to simulate loss of vision in virtual reality, participants could freely view omnidirectional natural scenes. This protocol allows the simulation of vision loss with an extended field of view (>80°) and studying the head's contributions to visual attention. The time-course of visuo-motor variables in our pure free-viewing task reveals long fixations and short saccades during first seconds of exploration, contrary to literature in visual tasks guided by instructions. We show that the effect of vision loss is reflected primarily on eye movements, in a manner consistent with two-dimensional screens literature. We hypothesize that head movements mainly serve to explore the scenes during free-viewing, the presence of masks did not significantly impact head scanning behaviours. We present new fixational and saccadic visuo-motor tendencies in a 360° context that we hope will help in the creation of gaze prediction models dedicated to virtual reality.

## Introduction

During visual tasks, we rely on peripheral information to explore our environment and on foveal information to analyse a region of interest in details (Larson & Loschky, 2009; Loschky et al., 2019). Studying the deployment of visual attention across our entire field of view is important for an understanding of visual perception past regular screen monitors, toward more natural everyday tasks, and visual attention modelling applications dedicated to omnidirectional contents (360° stimuli). It is only recently, with the advent of new virtual reality technologies, that research has started to focus on omnidirectional visual stimuli. In particular, in the context of gaze position prediction for compressing, storing and streaming applications (Sitzmann et al., 2017; Li et al., 2019). As a matter of fact, few saliency and saccadic models dedicated to 360 applications exist to date (Gutiérrez et al., 2018; Li et al., 2019). The role of our central and peripheral fields of view has been extensively studied via on-line simulations of visual field loss on screen. These studies share some experimental limitations, three are of particular importance to us:

• The extent of the peripheral field of view excited by a two-dimensional (2D) monitor is narrow (little of the retina is excited past the macula).
• The artificial mask in a gaze-contingent system is tied to one eye position or the average of the two.
• The use of body and head movements to observe a stimulus is severely limited.

We propose to remove these limitations through the use of a virtual reality head-mounted display (HMD), and study visuo-motor variables and their time-course with and without gaze-contingent masking. Modern HMDs stimulate 110° by 110° of field of view binocularly (in practice closer to 90° by 90°). Observers can use a significant portion of their peripheral field of view when planning saccades and exploring a scene. HMDs have a dedicated display per eye allowing to simulate visual field loss independently per view. Finally, because the display device and the eye-tracker are head mounted, participants can use their whole body to accomplish a visual task. In this new experiment combining a gaze-contingent protocol and virtual reality we study gaze, eye and head movements and the effect central and peripheral vision loss has on them. We will focus on comparing our results with on-screen experiments and on reporting visuo-motor biases during free-viewing of natural omnidirectional scenes.

### Head contribution to gaze movements

Owing to experimental imperatives, most eye tracking studies restrain or limit head movements and do not consider them during visual observation. However, it is important to consider the role the head plays in everyday life (Leigh & Zee, 2015, chapter 2; Fischer, 1924; Malinov et al., 2000; Einhäuser et al., 2007).

We can classify head movements into two categories: compensatory and synergistic. Compensatory movements stabilise gaze on a target while a scene is in motion. The vestibulo-ocular response will stabilize our gaze during short head motions (Einhäuser et al., 2007) most notably while we walk. It is based on a fast neural network allowing eye muscles to respond to vestibular signal with a low latency (≈16 ms; Barnes, 1979; Collewijn & Smeets, 2000). The vestibular system becomes less accurate during longer head movements; it is then superseded by the optokinetic response (Leigh & Zee, 2015, chapter 2; Robinson, 1981). It is slower to activate (≈75 ms) because it is induced visually by the scene moving on the retina (Gellman et al., 1990). In practice, this will translate to fixations during which head and eyes are in movements while the combined gaze is stable on a stimulus.

The second type of head movements is said to be synergistic (Einhäuser et al., 2007). Whereas the fixation field is defined as the area of the field of view where we are most likely to fixate only with our eyes, the practical field of fixation describes where our gaze is most likely to fall next using our head and eyes (Rötth, 1925). Studies pertaining to the fixation field are old (Asher 1898; Hofmann, 1925) and its span varies individually (Yamashiro, 1957; Von Noorden & Campos, 2002); however, we learn that, without head movements, saccades are made on average within 28° to 40° upward, 45° downward, 20° to 42° outward (temporal) and 35° to 46° inward (nasal). Head movements extend the field of view: saccades can be planned toward regions outside of it (Guitton & Volle, 1987; Von Noorden & Campos, 2002). There is a preference for short eye rotations completed by head movements (Janson et al., 1987; Malinov et al., 2000; Einhäuser et al., 2007; Freedman, 2008). The head accompanies the eyes, even during small gaze saccades (Ritzmann 1875; Bizzi et al., 1972; Einhäuser et al., 2007; Hu et al., 2017). Stahl described the eye-only-range (Stahl, 1999), an interval of saccade amplitudes within which head movements are less likely to occur. The *eye-only-range* is reported to be approximately 16° to 20° of eccentricity to the fovea (Stahl, 1999; Freedman & Sparks, 1997); This interval seems to be task dependent (Doshi & Trivedi, 2012) and was observed in laboratory settings where subjects were sitting (or strapped; Freedman & Sparks, 1997) and had to fixate bright target points.

Stahl (1999) has shown a linear relationship between head movements and the combined gaze saccade amplitude produced, showing that longer saccades elicit bigger head movements. In effect, eye-in-head movements tend to stay centred in the orbits. Bahill et al. (1975) has demonstrated that observers rarely produce eye movements greater than 15°, this result has been replicated in natural conditions (Einhäuser et al., 2007; Sullivan et al., 2015). Additionally, the head seems to contribute more during horizontal than vertical saccades (Fischer, 1924; Fang et al., 2015). Although rare, during oblique saccades, the head is particularly involved horizontally (Glenn & Vilis, 1992; Tweed et al., 1995; Freedman & Sparks, 1997).

### Visuo-motor biases during “natural” visual observation

By visuo-motor biases, we designate behavioral tendencies of fixation and saccade as measured through head, eye and the combined gaze. Mobile eye-tracking is used to study vision during natural tasks (Hayhoe & Ballard, 2005; Land, 2006; Kowler, 2011; Tatler et al., 2011; Lappi, 2016; Spratling, 2017), such as object manipulation (Pelz, 1995; Land et al., 1999), driving (Hayhoe et al., 2012; Lappi et al., 2013; Johnson et al., 2014), obstacle course (Franchak & Adolph, 2010), climbing (Grushko & Leonov, 2014), and so on (see Land, 2006; Lappi, 2016). In these conditions, the peripheral field of view is usually completely stimulated and movements can be unrestricted. In natural conditions, visuo-motor characteristics vary somewhat (Kowler, 2011; Lappi, 2016). Foulsham et al. (2011b) noticed that fixations are more scattered when participants observed a video of somebody walking outside compared with an individual actor of the action recorded. The horizontal bias observed on screens (Foulsham et al., 2008; Tatler & Vincent, 2009) is observed in natural conditions (Einhäuser et al., 2007; Foulsham et al., 2011b; Sullivan et al., 2015); although the rate of horizontal to vertical saccades varies according to the environment (Einhäuser et al., 2007) and the task (Sullivan et al., 2015). Foulsham et al. (2011b) described a horizon-centred bias where gaze is centred horizontally and vertically (see also Foulsham et al., 2008; Foulsham & Kingstone, 2010), as well as a tendency for eyes to stay centred in their orbit.

Studies observing eye and head movements during the viewing of omnidirectional contents in virtual reality report the same centre biases. Gaze is centred latitudinally around the horizon (*equator bias*; Sitzmann et al., 2017; Rai et al., 2017a; De Abreu et al., 2017; David et al., 2018; Xu et al., 2018); this tendency seems to depend on visual content (Anderson et al., 2020; Bischof et al., 2020). Gaze is also centred longitudinally (Rai et al., 2017a; David et al., 2018), probably because natural scenes often have a photographic point of interest; this may also be due to a starting exploration position shared among observers in experiments (Rai et al., 2017a; Xu et al., 2018; David et al., 2018). Head movements also display these tendencies (Corbillon et al., 2017; Wu et al., 2017; Xu et al., 2017). In virtual reality, eyes are observed to be centred in their orbit and saccades are rarely planned beyond 15° of eccentricities (Rai et al., 2017b; Sitzmann et al., 2017).

The current state of the literature tends to indicate that the observation of complex scenes is modulated by phases of local and global scanning during which visual attention is directed toward behaviours of exploration of the scene and fine analysis of regions of interest respectively (Tatler & Vincent, 2008; Godwin et al., 2014; Velichkovsky et al., 2019). Another way to see this dichotomy is as ambient and focal visual phases (Eisenberg & Zacks, 2016; Gameiro et al., 2017; Ehinger et al., 2018). During ambient phases, attention would be directed toward the content of the peripheral field of view to build a representation of the scene or to find new regions of interest to redirect gaze to; this is exploratory in essence. In contrast, during focal phases attention would be directed toward the fine analysis of central information to analyse one region of the scene in particular. Ambient phases are most notably measured at scene onset; they may serve to build a representation of the scene's content (Unema et al., 2005; Eisenberg & Zacks, 2016). Scene exploration is characterised by short fixations followed by long saccades, whereas an analysis of a region of interest is set apart by long fixations and short saccades (Eisenberg & Zacks, 2016). Ambient and focal processing is to be compared with the time-course of bottom-up and top-down processing of natural scenes. Visual attention appears to be guided by bottom-up processes immediately at scene onset, before transitioning to top-down processing for a short time, the rest of the viewing activity sees both processes interflow (Theeuwes et al., 2000; Connor et al., 2004; Delorme et al., 2004; Rai et al., 2016). Recent virtual reality (VR) studies (Solman et al., 2017; Bischof et al., 2020; Anderson et al., 2020) hint at the possibility that the head and the eyes could be controlled differently when exploring visual scenes. In a gaze-contingent study, Solman et al. (2017) demonstrated that the eyes would rather exploit the part of the field of view left visible, whereas head movements would serve more to make significant shifts in the content of the field.

Two particular saccadic biases are reported in the literature in relation to complex scene viewing. One is oriented toward the exploration of the scene and guides new saccades in the same direction as the preceding ones (saccadic momentum; Smith & Henderson, 2009, 2011); the other is related to the analysis of regions of interest, it is characterized by backward (possibly refixative) saccades (facilitation of return; Smith & Henderson, 2009). Both biases appear as modes of the distribution of saccade relative directions (see David et al., 2019 Figure 7): located at 0° (forward) and 180° (backward) of angle. When building dynamic models of visual attention (gaze prediction or saccadic models), we believe it is important to take these temporal dynamics into consideration, namely by weighting exploratory and analysing biases as a function of time, but also, when predicting gaze given the previous n seconds of scanpaths data, a model could benefit from inferring a current viewing phase (ambient or focal) and modify its saccade length and fixation duration distribution priors accordingly.

### Gaze-contingent masking

Gaze-contingent protocols have been used to study central and peripheral vision in a variety of tasks for close to 50 years (Duchowski et al., 2004; Aguilar & Castet, 2011). The principle consists in modifying a visual stimulus according to the current gaze position. Starting in the 1970s with the works of McConkie and Rayner (1975) and Rayner and Bertera (1979) with reading tasks, it has since been used in natural scene viewing by removing all peripheral or central information (van Diepen & d’Ydewalle, 2003; David et al., 2019), or low (Laubrock et al., 2013; Nuthmann, 2013; Nuthmann, 2014; Cajar et al., 2016b, 2016a) and high spatial frequencies (Loschky & McConkie, 2002; Loschky et al., 2005; Foulsham et al., 2011a; Laubrock et al., 2013; Nuthmann, 2013; Nuthmann, 2014; Cajar et al., 2016b, 2016a), or colour (Nuthmann & Malcolm, 2016).

Gaze-contingent masks affect global scene processing and saccade planning as reflected by an increase in fixation duration (Cornelissen et al., 2005; Laubrock et al., 2013; Nuthmann, 2014; Nuthmann & Malcolm, 2016; Cajar et al., 2016b). In particular, removing peripheral vision impairs global scene processing as seen through an increase in average initiation and scanning times during visual search (Nuthmann, 2013; 2014). It also affects saccades: they target areas where the best visual information was available when they were programmed (Foulsham et al., 2011a). With central masking, saccade amplitudes increase to target areas past the mask and return saccade rates increase as observers try to analyse foveally objects of interest in spite of the loss of vision (Henderson et al., 1997; Cornelissen et al., 2005; David et al., 2019). Conversely, by simulating a peripheral loss of vision, saccades decrease in amplitude as they target locations within the central area left unmodified; mask's effect on saccade amplitudes shows a clear correlation with the radius of the mask (Loschky & McConkie, 2002; Loschky et al., 2005; Foulsham et al., 2011a; Nuthmann, 2013, 2014; Cajar et al., 2016a; Geringswald et al., 2016; David et al., 2019). Masking also affects head and eye movements planning (Solman et al., 2017). Watson et al. (1997) demonstrated that visual search in a virtual environment was hardly hampered by low resolution visual stimulations in the periphery.

Gaze-contingent multiresolutional displays (GCMRDs; Reingold et al., 2003; Duchowski & Çöltekin, 2007) take advantage of the uneven sensitivity to spatial frequencies (Loschky & McConkie, 2000, 2002) or colour (Duchowski & Çöltekin, 2007) of the visual system as a function of eccentricity to the fovea to degrade visual information without notice from the observers. The goal of a GCMRD is to decrease the amount of information needed to be processed or transmitted (Reingold et al., 2003) without interfering with the user's experience or comfort. This use case is an example of a moving window where visual information is best at the gaze point and degraded away from it. Applied specifically to virtual reality applications it is referred as *foveated rendering* (Guenter et al., 2012; Patney et al., 2016). The first commercial devices implementing foveated rendering appeared in the 1990s (Zeevi & Ginosar, 1990; Fernie, 1995). Yet studies have focused on the detection of peripheral modifications (Geisler & Perry, 1998; Fortenbaugh et al., 2008; Guenter et al., 2012; Patney et al., 2016; Albert et al., 2017). To the best of our knowledge, no study has investigated the impact of central or peripheral vision loss on visuo-motor behaviours with a gaze-contingent system implemented in an HMD. We propose to implement just that and extend the literature on visual field losses simulated with an extended field of view and the use of head and body movements.

### The present research

In this study, we aim to investigate how observers use their eyes and head when viewing omnidirectional scenes. Participants were asked to freely view 360° scenes with a central or peripheral gaze-contingent mask, or without a mask. A central mask, removed visual information centered on the gaze position to an extent of six or height°; contrariwise, peripheral masking only preserved a disk of visual information (4° or 6°) centered on the gaze point. The use of gaze-contingent masking is meant to shed light on the role of peripheral and central visions, it is also implemented as a mean to replicate on-screen studies segregating fields of view in the same manner (David et al., 2019). The use of a VR headset allowed to measure eye, head and gaze movements; therefore our hypotheses cover all three types of movements.

Considering the effect of gaze-contingent masks on scene analysis and saccade planning we would expect the average fixation duration to increase in presence of masks. Nonetheless, we observed previously in a pure free-viewing task (David et al., 2019) that fixation duration decreased without central vision may be because there was no incentive to analyse finely the scene and because participants made short fixations on targets of interest before their attention was grasped by peripheral information. As noted by Henderson et al. (1997): “data suggests that the absence of foveal information leads the eyes to move along to a new (currently extrafoveal) source of information as quickly as possible” (p.334). Our previous study (David et al., 2019) simulating peripheral vision loss on a regular monitor showed a decrease in average fixation duration with bigger masks (3.5° and 4.5° of radii), but averages are similar or above the no-mask condition with smaller masks (1.5° and 2.5°). Therefore, considering our choice of peripheral mask radii (4° and 6°), we expected fixation durations to decrease when peripheral information was removed.

We expected eye and gaze saccade amplitudes to increase with central vision loss as participants plan new fixations beyond the mask (Cajar et al., 2016b, 2016a; David et al., 2019). Kollenberg et al. (2010) demonstrated that participants fitted with an HMD stimulating only 25° of field of view (12.5° radius) made fewer eye rotations, head movements amplitude increased to compensate for it. Thus, we may observe an increase in head movement amplitudes in the peripheral mask conditions, as well we expected a decrease of eye movement amplitude linked with peripheral masking (Loschky & McConkie, 2002; Loschky et al., 2005; Foulsham et al., 2011a; Laubrock et al., 2013; Nuthmann, 2013; Nuthmann, 2014; Cajar et al., 2016a, 2016b; David et al., 2019).

The horizontal bias is fairly stable de spite simulated vision losses (Foulsham et al., 2011a; David et al., 2019). We expected to observe it expressed by eye and gaze movements; per the literature we expected head movements to contribute mostly horizontally. During scene viewing on screen, we reported on two saccadic relative direction biases (David et al., 2019). We expected more return saccades when central information is missing (Henderson et al., 1997; Cornelissen et al., 2005; David et al., 2019) and more forward saccades when peripheral information is missing (David et al., 2019). There is no information in the literature regarding the relative directions of saccades in an omnidirectional environment. We expected eye and gaze movements to express the two biases presented. We hypothesized that relative head motion will contribute to the overall gaze and therefore will exhibit the same tendencies. In a second time we analyse the time-course variations of the aforementioned variables. As per the literature, we expected scene viewing to start with ambient processing behaviour via short fixations and long saccades. Average fixation durations should decrease and average saccade amplitudes should increase after scene onset as ambient and focal processes are interleaved. As was observe in VR previously (David et al., 2020), we expected head movements rotations to decrease at first and progressively increase thereafter. Overall we expected the effects of vision losses to be reflected more strongly through eye rotations. We hypothesized that head movements would play a coarser role of exploration in gaze movements, whereas eye rotations would portray a finer control of scene analysis.

## Method

### Participants

Fifty participants were recruited for this experiment via a mailing list reaching mostly students of Nantes University (32 women; mean age, 22.5 years old; minimum, 19, maximum, 49). Normal vision was tested with a Monoyer test and normal color perception with the Ishihara color blindness test. We did not test stereo-depth perception because our stimuli are not stereoscopic. We measured the observers’ interpupillary distance and adapted the distance between the HMD's lenses accordingly to obtain the best viewing conditions (Best, 1996). We finished by determining the dominant eye with the Dolman method (Cheng et al., 2004). All participants gave their written consent before beginning the experiment and were compensated for their time. This experiment conformed to the Declaration of Helsinki and was approved by the Ethics Committee of the French Society of Ophthalmology (IRB 00008855 *Société Française d’Ophtalmologie* IRB#1).

### Apparatus

Stimuli were displayed in a virtual reality headset HTC VIVE (HTC, Valve corporation) retro-fitted with an eye tracker system (SMI, SensoMotoric Instrument). Within the HMD are two viewports, each half of the total display resolution (1,800 × 1,200 pixels) and representing approximately 90x90° of field of view combined. The headset's frame rate is 90 Hz, the eye tracker samples gaze at 250 Hz. The display and gaze processing computer runs an Nvidia GTX 1080 GPU and an Intel E5-1650 CPU.

We implemented a custom procedure to validate the calibration performed by the eyetracker, because the vendor's implemented calibration and validation procedures do not allow for automatic checks of the accuracy (instead a validation accuracy is displayed directly on screen). We implemented our validation protocol so that it would shorten the procedure when possible: if the recorded gaze was within 2.5° of the target point for 200 ms, it would cut to the next validation target point. Otherwise, the threshold for a passing accuracy was 3° of average dispersion. The effective calibration was more precise than that (the vendor reports a minimum accuracy of 0.2°), but our validation procedure was meant to quickly test if it was within our accepted accuracy level, without tiring the participants with long and repeated validation phases.

The maximum latency from the movement of an eye to the update of the gaze-contingent mask on screen is less than 30 ms. This “worst-case scenario” latency is obtained by adding up the latency of all components of the gaze-contingent system: display refresh rate, eye tracking sampling rate and eye tracking processing time. The latency is critical when implementing a gaze-contingent protocol. For instance, because the lag between a mask's position and the true gaze position means that participants may partially perceive with their central vision when it should be occluded. A maximum of 30 ms is acceptable considering saccadic suppression (Ilg & Hoffmann, 1993; Thiele et al., 2002), our choice of mask sizes, and the fact that the mask can only lag in regard to an eye movement, not the combined head and eye movements.

### Stimuli

**Figure 1.**
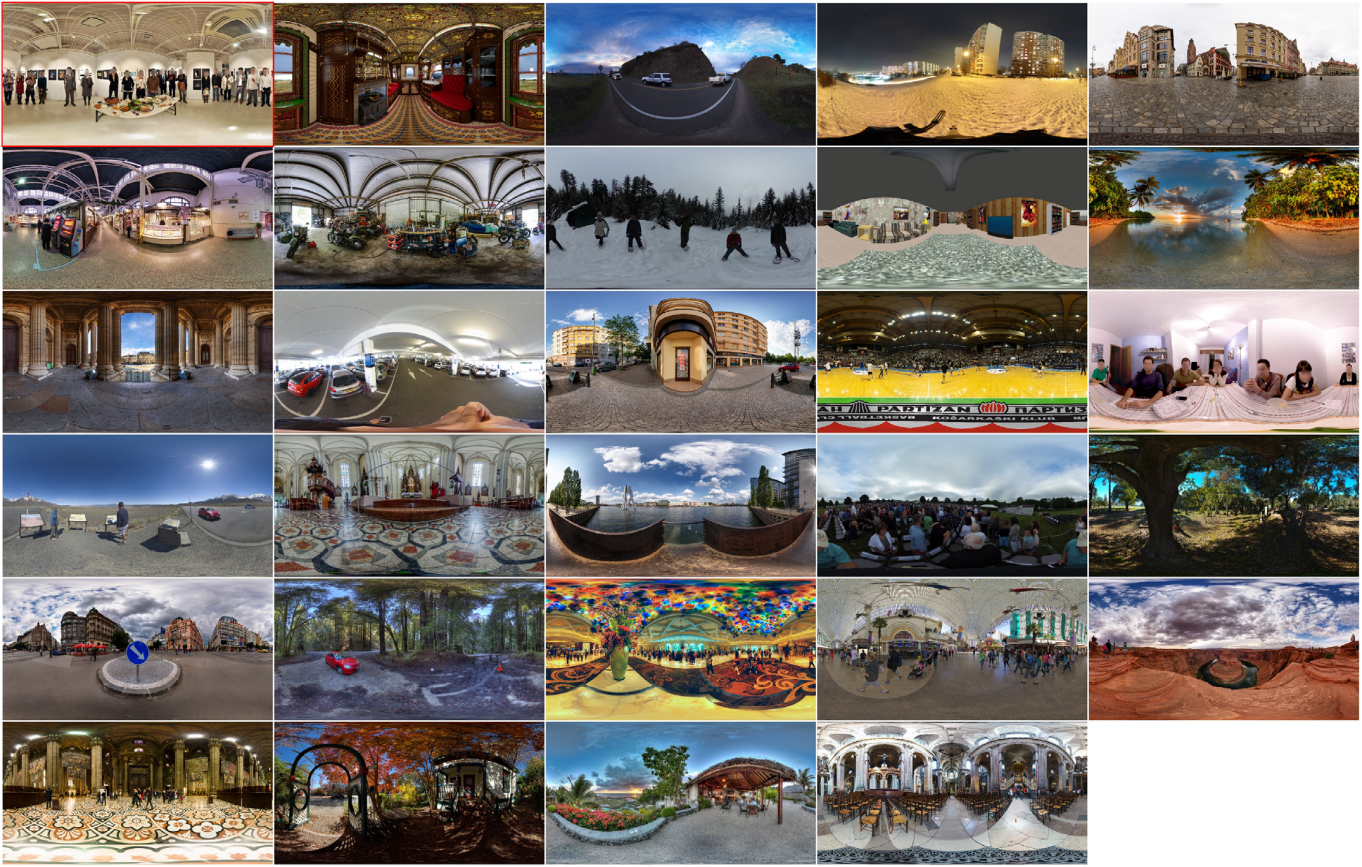
The 29 omnidirectional images were used in this experiment, the first one (red border) was part of the training phase.

The omnidirectional stimuli dataset is made of 29 images and 29 videos (indoor and outdoor scenes) borrowed from the training datasets (Rai et al., 2017a; David et al., 2018) of the Salient360! benchmark (Gutiérrez et al., 2018). All stimuli are in 4K resolution (3,840 × 1,920 pixels), not stereoscopic, and in colour. They are stored on disk as equirectangular two-dimensional projections and back-projected on a sphere during the experiment by a GPU routine. The content sphere followed the headset's translations during the experiment to ensure that observers were always located at the centre of the omnidirectional scene. One image and one video were set aside and used in a training and habituation phase. In this study we are solely interested in the analysis of the static stimuli (Figure 1).

### Experimental design

We implemented a gaze-contingent paradigm in a virtual reality headset to study visuo-motor behaviours during the free-viewing of omnidirectional stimuli. The omnidirectional content displayed in the HMD was updated 90 times per seconds with gaze positions sampled by the eye tracker. Gaze-contingent masks were drawn in an alpha-blending operation and appeared as grey circles with smoothed edges blending the mask with the stimulus, reducing the effect of sharp mask edges on visual attention (Reingold & Loschky, 2002). The position of the mask on the left display was updated with the position of the left gaze, right gaze served to update the right display.

**Figure 2.**
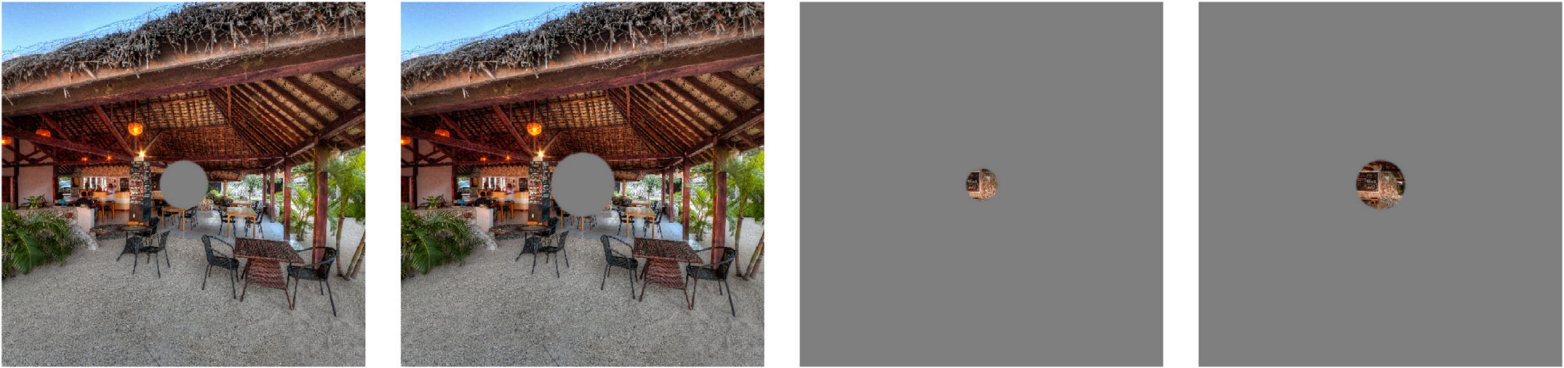
Masking conditions are presented here in a viewport measuring 90° by 90° of field of view. Radii are proportionally accurate. From left to right: central masks of 6° and 8° of radius, peripheral masks of 4° and 6° of radius.

To select mask radii adapted to the constraints and limitations of the apparatus, we run a prestudy (5 subjects; section A.1, Appendix). Consequently, we chose central masks of 6° and 8° of radius, and peripheral masks of 4° and 6° of radius. The radii chosen in this study are larger than usually encountered in gaze-contingent studies (e.g., Cajar et al., 2016a; Loschky & McConkie, 2002). Nevertheless, we have to consider that the total field of view excited here is considerably extended (≈90° × 90°) compared with gaze-contingent experiments on screen (e.g., in a previous study the total field of view was 31.2° by 17.7°; David et al., 2019). The final protocol has four mask modalities and a control condition without masking (Figure 2).

**Figure 3.**
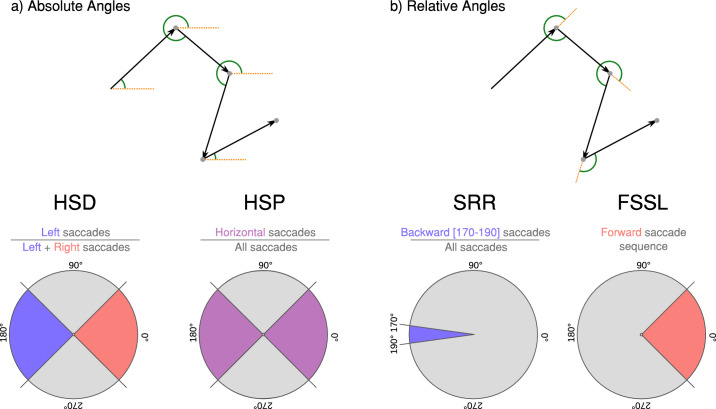
(a) Absolute angles appear in green between a saccade vector (black arrows) and the horizontal axis (orange dashed lines). The horizontal saccade directionality (HSD) reports the proportion of left-directed saccades within horizontal saccades; the horizontal saccade percentage (HSP) measures the proportion of horizontal saccades among all saccades observed. (b) Relative angles (green arcs) are angles between two saccades vectors (black arrows and orange dashed lines). The saccadic reversal rate (SRR; Asfaw et al., 2018) measures the number of backward saccades falling between 170${}^{\circ }$ and 190${}^{\circ }$ as a proportion of the total amount of saccades. The forward saccade segment length (FSSL) reports on the length distribution of consecutive saccades directed approximately in the same relative forward direction.

### Procedure

Participants were told to freely observe omnidirectional stimuli. Sitting on a swivel chair, they could rotate three full turns before being hindered by the HMD's cable. We prevented observers from standing because they were not aware of their surroundings while wearing the headset and may have collided with walls and furnitures.

Participants started with an eye tracker calibration (five points) and validation phase (nine points). Following that, they would observe a training image and video for a minute each in order to get used to the virtual reality settings and material without vision loss. During this training phase, they were encouraged to look all around them and experience the omnidirectional nature of the protocol.

A validation procedure was triggered every seven trials. If the average gaze distance to a target validation point was detected to be more than 3°, a new phase of calibration and validation would begin. Successful validation phases showed an acceptable mean degrees of dispersion over all nine validation dots (95% confidence interval (CI) 1.9–1.94).

Participants observed 56 stimuli for 20 seconds in a random order of presentation. Omnidirectional contents were offset longitudinally according to the head rotation at the start of the trial, so that all participants started viewing the stimuli at the same longitude. Participants observed each stimulus only once, presented in one of the masking conditions in a random order of stimulus per masking condition (total: 56 trials). They experienced the three masking conditions approximately the same number of times. We counterbalanced trial conditions so that a stimulus would be viewed with each mask the same number of time in the final data set. The experiment lasted less than 40 minutes, with a resting period midway through.

### Data preparation

In this study we dissociated movements of the head and the eyes (eye-in-head), and we will refer to the combination of both as the *combined gaze* (eye-in-space; Lappi, 2016; Larsson et al., 2016; Hessels et al., 2018). Raw eye data were received from the eye tracker as normalized positions on the two-dimensional viewport plane for each eye. Head rotation data are the tracking data from the VR headset; it is important to note that head rotations, as reported in this study, are influenced by movements of the neck, as well as the torso and the chair the participants sat in.

Eye positions on the two-dimensional displays were projected on a unit sphere to obtain 3D eye rotation vectors. That same 3D eye data was added to the camera rotation data (quaternion) to obtain gaze directions in the 3D world (eye-in-space), then transformed to a position on the sphere (longitudes and latitudes). Data processing was achieved with the help of a in-house toolbox developed for the Salient360! benchmark (David et al., 2018; Gutiérrez et al., 2018).

In our analyses, we considered eye movements from the dominant eye only. Saccades and fixations were identified with a velocity-based parsing algorithm (Salvucci & Goldberg, 2000) on the basis of gaze movements (eye-in-space). Gaze velocity was defined in degrees per millisecond as the orthodromic distance between two gaze samples divided by their time difference. The gaze velocity signal was then smoothed with a Gaussian filter (σ=5 samples) to decrease the effect of noise. We parsed gaze movements (and not eye movements alone) in consideration for the compensatory movements (vestibulo-ocular and opto-kinetic responses) of the head during which gaze may be still whereas head and eyes are in movement. We chose a velocity threshold of 100°/s to separate saccades from fixations.

We removed from our dataset 95 trials missing more than 20% of either left or right eye samples, eye tracking loss meant that a participant could observe the scene within one display with a mispositioned mask., 7354 “short fixations” (<80 ms, 8.73%) and 42 “long fixations” (>2,000 ms, 0.05%) from the dataset. In the first case we considered 80 ms to be the minimum amount of time needed to analyze a visual stimulus and plan a new saccade (Salthouse & Ellis, 1980; Manor & Gordon, 2003; Leigh & Zee, 2015); in the second case, we considered “long fixations” to account for cognitive processes independent from the task (Inhoff & Radach, 1998). The final dataset is composed of 76,815 fixations and 73,186 saccades (these figures concern only static stimuli trials).

In a previous study (David et al., 2019), we showed the importance of studying saccade directions. We defined absolute saccade directions as the angle between a saccade vector and the horizontal axis, and relative directions as the angle between two consecutive saccade vectors. Considering head and gaze movements, we obtained angles between saccades by transforming fixation positions (longitudes and latitudes) from a 3D sphere to a two-dimensional plane with a Mercator projection (Equation 1). A Mercator projection is conformal (preserves angle measurements), we computed absolute and relative directions between two-dimensional vectors on a Mercator plane because this method is simpler than calculating angles of vectors on a sphere in 3D space. On the display, eye movement directions were computed as the angle between two-dimensional eye movement vectors. Eye movement amplitudes during saccades are the Euclidean distance between fixation centroids on the displays; head and gaze amplitudes are the orthodromic distance between fixation centroids on the sphere.

(1) $$\begin{array}{c} \begin{array}{ccc} \lambda_{mercator} & = & \lambda \\ \phi_{mercator} & = & \log\left(\tan\left(\frac{\pi }{4}+\frac{\phi }{2}\right)\right) \end{array}\; \end{array}$$

Where λ is a longitude (-π<λ<π) and ϕ a latitude ($-\frac{\pi }{2}<\phi <\frac{\pi }{2}$). Previously, we transformed absolute and relative angles into measures of ratios to check the nature of the saccade direction biases with LMMs (David et al., 2019). We first defined the horizontal saccade directionality (HSD), measuring the proportion of leftward directed saccades ([135°–235${}^{\circ }$]) among horizontal saccades ([135°–235${}^{\circ }$] and [315°–45${}^{\circ }$]); the horizontal saccade percentage (HSP) is the number of horizontal saccades ([135°–235${}^{\circ }$] and [315°–45${}^{\circ }$]) divided by the total saccade count in a trial. Finally, we measure the saccadic reversal rate (Asfaw et al., 2018) for a precise look at backward saccades: the number of saccades directed in a [170°–190${}^{\circ }$] interval relative to the previous saccade direction, divided by the total number of saccades in a trial. To measure a hypothesised exploratory behaviours occurring across several saccades (scanning line pattern) we calculate forward saccade segment lengths (FSSL): considering one trial's scanpath, we identified segments comprised of successive saccades going approximately in the same direction (90° window forward). We recorded the number of saccades making up each segment (length). This operation was accomplished for the tracked head, eye and combined gaze data, on the basis of the saccadic relative angle data (computed on the basis of the combined gaze data).

## Analyses

We relied on linear mixed models (LMMs) to estimate statistical differences between mask conditions in regard to our choice of dependent variables. LMMs account for random experimental effects such as response differences between randomly sampled subjects or subjects’ idiosyncratic reactions to stimuli. For each measure we tested two hypotheses: 1) vision loss data compared with control results in a significantly different behavior; 2) the importance of vision loss (according to mask radius) influences visuo-motor behaviours. Four comparisons were planned with contrasts (control data vs. central mask, control vs. peripheral; central 6° vs. central 8°, peripheral 4° vs. peripheral 6°) using the *lme4* package (Bates et al., 2014) for R (The R Project for Statistical Computing; R Core Team, 2018). Second, we report on exploratory behaviours with the FSSL measure. In a third set of analyses, we visually study time variations of fixation duration, saccade amplitude and backward saccade rates. Finally, an analysis of fixation centre biases are reported in section A.3 of the Appendix.

The effects of stimuli and subjects were accounted for in the LMMs as random intercepts. We chose to report the following values from the LMMs: b-value (estimated difference between means), SE (estimated standard error) and t-value. As noted by Cajar et al. (2016b), and Nuthmann and Malcolm (2016): as sample sizes increase the t distribution converges towards the normal distribution, therefore we can consider absolute t-value above 1.96 to be significant (Baayen et al., 2008). The b-values are here reported as effect sizes that includes mixed effects. We log-transformed the measures of head, eye and gaze amplitudes, as well as fixation duration to improve the normality of the residuals and the reliability of the models. Log-transformed variables are presented on a logarithmic scale in the figures that follow.

### Fixation duration

**Figure 4.**
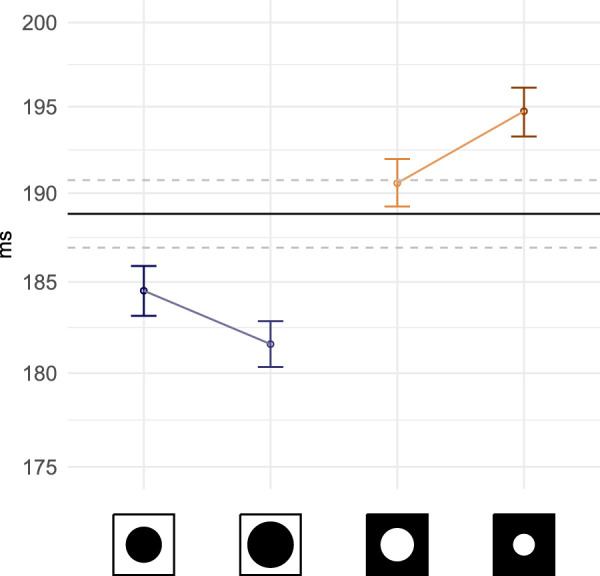
Average and 95% CI of fixation durations calculated across subjects and stimuli (on a log-scale). The X axis labels have been replaced with icons representing mask types and radii, from left to right: central mask 6°, central 8°, peripheral 6°, peripheral 4°. Mask conditions are ordered by increasing surface masked. The control condition is present as a black line crossing the plots horizontally (mean is shown as a solid black line, dashed lines report 95% CI).

Fixation duration is an indicator of visual attention processing (Nuthmann et al., 2010): analysis of central information (van Diepen & d’Ydewalle, 2003; Nuthmann & Malcolm, 2016) and planning of next saccades (Cajar et al., 2016a). Without masking, participants made somewhat shorter fixations overall (95% CI, 190.6 – 194.4) than what was observed on screen during free-viewing in the same condition (95% CI, 275.9 – 281.5; David et al., 2019). Our analysis (Figure 4) shows that trials with central masks reduced average fixation durations (b=-0.04,SE=0.01,t=-7.24), whereas a peripheral mask increased them (b=0.03,SE=0.01,t=6.17). In each of the conditions, mask size influenced the duration of fixations. The larger the central window, the shorter the duration of fixation (b=-0.02,SE=0.01,t=-3.07); during peripheral masking conditions, the smaller the window the longer the duration of fixation (b=-0.03,SE=0.01,t=-4.88).

### Saccade amplitude

**Figure 5.**
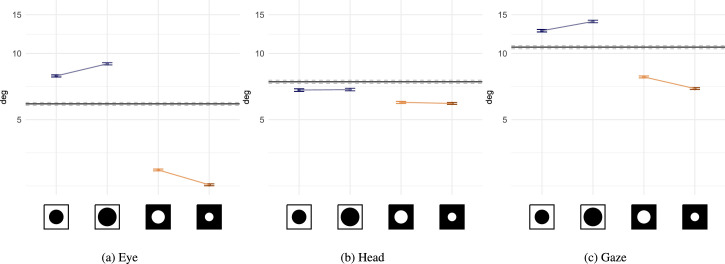
Average and 95% CI of the head, eye and combined movement amplitude during saccades calculated across subjects and stimuli (on a log-scale). For a more detailed view, we present in Figure A.7 density distributions of gaze, eye and head movements as a function of the amplitude of the motion during saccades. The X axis labels have been replaced with icons representing mask types and radii, from left to right: central mask 6°, central 8°, peripheral 6°, peripheral 4°. The control condition is present as black lines crossing the plots horizontally (mean is shown as a solid black line, dashed lines report 95% CI).

The distribution of eye movement amplitudes (Figure A.7b, Appendix) without a mask is similarly shaped to the one observed on screen (David et al., 2019). The difference being that its mode is almost twice as big (≈2 in David et al., 2019, compared with ≈4 in the present study). Head movements were on average longer than eye movements; as expected, they significantly participated in the combined gaze saccade made to navigate the 360° scenes (Von Noorden & Campos, 2002; Freedman, 2008). As expected from past research, eye rotations (Figure 5a) increased in amplitude when central information was masked (b=0.35,SE=0.01,t=49.29) and decreased when peripheral information was removed (b=-0.78,SE=0.01,t=-118.23). The mask radius influenced saccade amplitudes in both condition, average amplitude increased with bigger central mask (b=0.12,SE=0.01,t=15.22) and with bigger window (b=0.16,SE=0.01,t=22.54). We observed a significant decrease of head rotations in case of gaze-contingent masks (Figure 5b), with a greater effect in case of peripheral masking (peripheral: b=-0.26,SE=0.01,t=-35.76: central: b=-0.09,SE=0.01,t=-10.51). The size of this effect appears globally stable whatever the mask size, with no effect according to the central mask radius (b=0,SE=0.01,t=0.01), and a weak effect according to the size of peripheral mask (b=0.03,SE=0.01,t=3.93). We see the that the density distribution of head movement amplitudes (Figure A.7c, Appendix) is bimodal in masking conditions. We posit that the presence of a mask decreased the probability for an observer to use their head while analyzing a region of interest to decrease changes in the field of view, which exacerbated the difference between ambient and focal behaviors in head movements. The effect of gaze-contingent masking on gaze amplitude is a cumulative effect of eye and head movements. We observed similar tendencies as eye movements alone (Figure 5c). Peripheral masking resulted in longer saccades (b=0.22,SE=0.01,t=25.99), whereas peripheral masking resulted in shorter saccades (b=-0.38,SE=0.01,t=-53.77). Bigger central masks elicited even longer saccades (b=0.09,SE=0.01,t=9.91), same was observed with peripheral masks (b=0.13,SE=0.01,t=18.44).

### Absolute saccade direction

**Figure 6.**
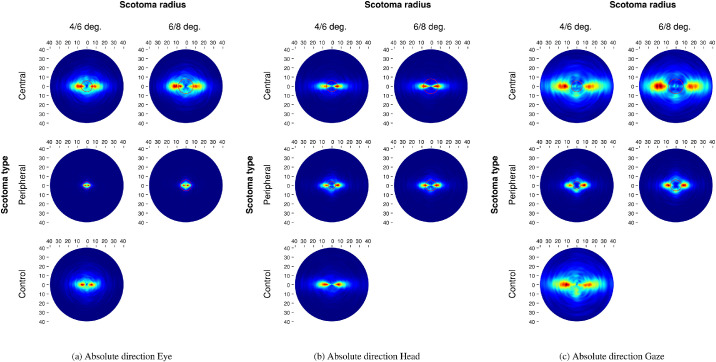
Joint distribution of saccade amplitude and absolute direction as a function of masking condition. The red circles represent the mask radius.

**Figure A.5.**
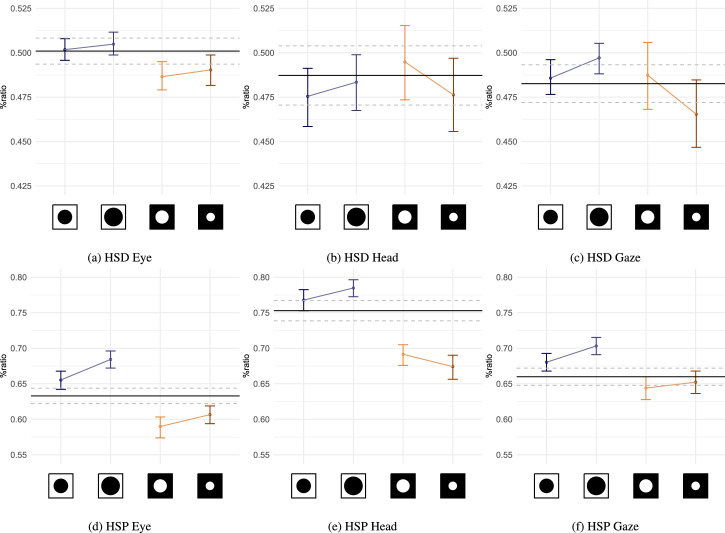
Average and 95% CI of HSD and HSP measures of absolute saccade direction calculated across subjects and stimuli. The X axis labels have been replaced with icons representing mask types and radii, from left to right: central mask 6°, central 8°, peripheral 6°, and peripheral 4°. The control condition is present as black lines crossing the plots horizontally (mean is shown as a solid black line, dashed lines report 95% CI).

Figure 6 shows the joint distributions of saccade amplitudes and absolute directions. Viewing without a mask, head movements were predominantly horizontal (Figure 6a). While eye movements were also mostly directed horizontally, they showed some variance in particular downward, which shows in the resulting combined gaze data (Figure 6c). Within the display screen, the horizontal saccade ratios (HSD; David et al., 2019; Figure A.5a, Appendix) show that participants experiencing central masking produced as many saccades directed to the left as to the right similarly to the no-mask trials (b=0,SE=0,t=0.58). Presence of a peripheral mask slightly decreased the number of leftward saccades (b=-0.01,SE=0,t=-2.52). Mask sizes did not affect further the horizontal distribution of saccades (central: b=0,SE=0,t=0.7; peripheral: b=0,SE=0.01,t=-0.63). The horizontal to vertical saccade ratios (HSP, David et al., 2019; Figure A.5d, Appendix) shows an increase in horizontal saccades with central masks (b=0.04,SE=0.01,t=5.89), but a decrease with peripheral masks (b=-0.03,SE=0.01,t=-5.17). This effect was modulated by mask radii: the proportion of horizontal saccades increased with mask sizes in the case of central masking (b=0.03,SE=0.01,t=3.86), and decreased as peripheral masks grew in radius (b=-0.02,SE=0.01,t=-2.2). HSD measures of head movements (Figure A.5b, Appendix) show no effects linked to central (b=-0.01,SE=0.01,t=-0.78) or peripheral (b=0,SE=0.01,t=-0.13) masking. Variation in mask sizes did not have any effect either (central: b=0.01,SE=0.01,t=0.65; peripheral: b=0.02,SE=0.02,t=1.21). HSP measures (Figure A.5e) report a slight increase in horizontal head movements during saccades for central mask trials (b=0.02,SE=0.01,t=3.41) and a stronger decrease during peripheral trials (b=-0.07,SE=0.01,t=-9.09). Mask sizes had a small effect on HSP as more horizontal head rotations are produced with the biggest central (b=0.02,SE=0.01,t=2.4) and peripheral (b=0.02,SE=0.01,t=2.12) masks. When head and eye rotations are combined no particular effect of masking was observed on the horizontal distribution of saccades (HSD, Figure A.5c, Appendix) (central: b=0.01,SE=0.01,t=1.54; peripheral: b=-0.01,SE=0.01,t=-0.56). Likewise, participants showed no significantly different behaviour as a function of mask size (central: b=0.01,SE=0.01,t=1.7; peripheral: b=0.02,SE=0.01,t=1.61). The horizontal to vertical saccade ratios (HSP, Figure A.5f, Appendix) varied significantly when central visual information was masked (b=0.03,SE=0.01,t=5.33). A bigger central mask elicited even more horizontal saccades at the expense of vertical exploration (b=0.02,SE=0.01,t=3.44). No such effects were observed in presence of peripheral masks (b=-0.01,SE=0.01,t=-1.46) of any radii (b=-0.01,SE=0.01,t=-0.68).

### Relative saccade direction

**Figure 7.**
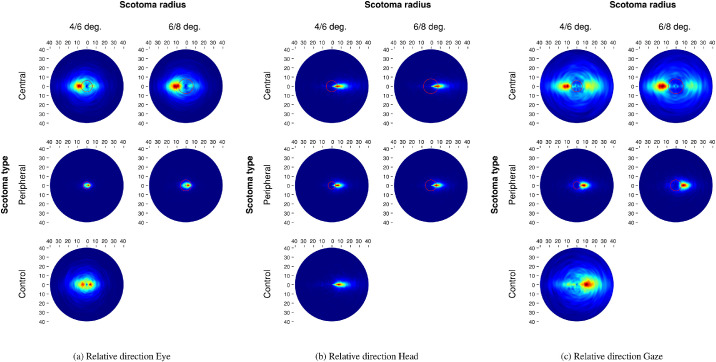
Joint distribution of saccade amplitude and relative direction as a function of masking condition. The red circles represent the mask radius.

The saccadic reversal rate (SRR; Asfaw et al., 2018) measures the proportion of backward saccades directed precisely in the direction of a fixation at t-1, these saccades can be characterized as return saccades. In the control condition, observers made almost as many backward and forward eye movements (Figure 7a), contrary to results obtained on screen (David et al., 2019) where we observed more forward saccades. The combined gaze relative saccade directions observed in this VR experiment approximate on-screen results because of head movements being almost exclusively directed forward (Figure 7b). Thus, in normal viewing conditions participants make more forward saccades thanks to head movements. In contrast, eye movements are characterized by backward and forward direction biases.

**Figure A.6.**
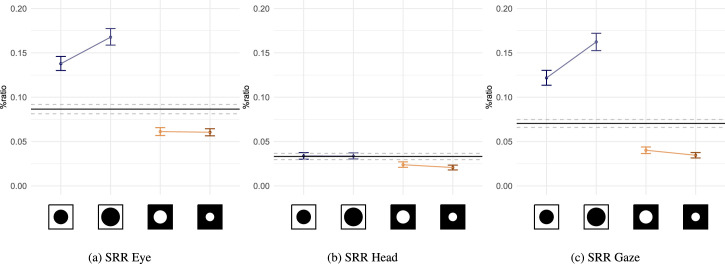
Average and 95% CI of saccadic reversal rates (SRR) of relative saccade direction calculated across subjects and stimuli. The X axis labels have been replaced with icons representing mask types and radii, from left to right: central mask 6°, central 8°, peripheral 6°, peripheral 4°. The control condition is present as black lines crossing the plots horizontally (mean is shown as a solid black line, dashed lines report 95% CI).

**Figure A.7.**
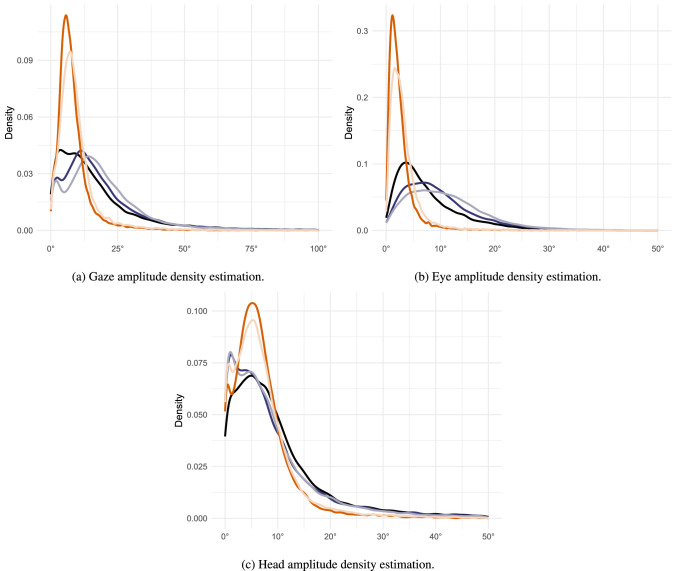
Figures A.7a, A.7b and A.7c show density estimations of saccade distributions as a function of gaze, eye and head movement amplitudes. Gaze movement amplitudes (Figure A.7a) are presented with a maxima of 100°. Colour legend: 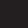 no-mask, 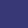 central 6°, 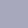 central 8°, 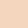 peripheral 6°, and 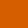 peripheral 4°.

Lacking central vision resulted again in an increase in the backward saccade rate (Figure A.6a, Appendix) as observers tried to analyze objects of interest in spite of a central mask (b=0.07,SE=0,t=14.6), a bigger central mask exacerbated this effect (b=0.03,SE=0.01,t=5.34). Conversely, the presence of a peripheral mask decreased the return saccade rate (b=-0.03,SE=0,t=-9.45), mask sizes showed no effect (b=0,SE=0,t=0.26). Looking at head movements, we notice a low SRR for the baseline no-mask trials (*Mean*: 3.3%, *SD*: 2.9%; Figure A.6b, Appendix). Central masking did not impact head movements (b=0,SE=0,t=0.38), whatever the mask sizes (b=0,SE=0,t=-0.14). In contrast, a peripheral mask decreased further the rate of backward saccades (b=-0.01,SE=0,t=-5.81), but the mask size had no effect on this result (b=0,SE=0,t=1.54). Considering the combined gaze movements, participants produced more backward saccades (Figure A.6c, Appendix) when central vision was removed (b=0.07,SE=0,t=15.3). This effect increased with the bigger mask size (b=0.04,SE=0.01,t=6.81). In contrast, lack of peripheral vision reduced the proportion of backward saccades (b=-0.03,SE=0,t=-14.33), the smaller mask slightly reduced this decrease (b=0.01,SE=0,t=2.41).

### Exploratory behavior beyond two saccades

We propose to measure the average length of sequences of saccades produced in approximately the same direction as a previous saccade (forward saccade segment length, FSSL). The goal of this analysis is to evaluate if there exists a strategy of exploration observable as consecutive motions of the head or the eyes continuing in the same general direction (scanning pattern). Low backward saccade rates of head movements measured in the preceding section hinted at this behaviour, but SRR only involves two saccades in its calculation. Seeing as we are interested in behavioral differences between head and eye movements in particular, we used LMMs comparing the type of movements (head, eye, combined gaze) rather than mask types. These LMMs also take into account the random effect of the stimuli and the subjects (as random intercepts). We removed data regarding the last half second of each trial, because the abrupt end potentially interrupted segments.

**Figure 8.**
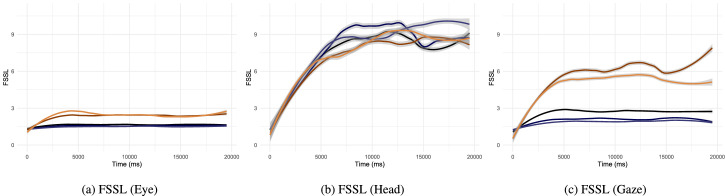
Mean and 95% CI of forward saccade segment length (FSSL) as a function of viewing time. A higher FSSL value means that a saccade at that point in time was on average part of a longer sequence of saccades travelling in the same direction. Colour legend: 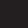 no-mask, 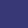 central 6°, 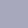 central 8°, 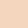 peripheral 6°, 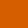 peripheral 4°.

Without a gaze-contingent mask, participants made on average longer sequences of movements in the same direction with their head compared to with their eyes (b=2.42,SE=0.03,t=73.78) or their combined gaze (b=2.06,SE=0.03,t=60.74). The difference between the eye and the combined gaze movements was negligible (b=-0.36,SE=0.03,t=-14.23). The same effect appeared during central mask trials. Head FSSL were on average higher than eye's (mask 6°: b=2.39,SE=0.03,t=77.02; 8°: b=2.5,SE=0.03,t=81.18) or the combined gaze (mask 6°: b=2.21,SE=0.03,t=70.02; 8°: b=2.36,SE=0.03,t=75.62), and the differences between these last two were small (mask 6°: b=-0.18,SE=0.02,t=-7.73; 8°: b=-0.14,SE=0.02,t=-6.23). In contrast, peripheral masking resulted in a smaller increase in head average sequence lengths compared to the combined gaze, than was observed with central masking (mask 4°: b=0.68,SE=0.05,t=14.21; 6°: b=1.11,SE=0.05,t=24.06). This is also true when comparing head with eye FSSL (mask 4°: b=2,SE=0.04,t=46.49; 6°: b=2.09,SE=0.04,t=49.36), this is the result of a general increase in forward motions (cf. SRR results in the previous section). FSSL of eye data are lower than the combined gaze (mask 4°: b=-1.32,SE=0.04,t=-32.94; 6°: b=-0.99,SE=0.04,t=-26.08). The time-course of FSSL (Figure 8) shows that during central masking and no-mask trials FSSL values were low and varied little, as far as the eye and the combined gaze movements are concerned. In contrast, FSSL of head movements increased in all masking conditions over a 5-second period after trial onset, and reached segment lengths of 8.7 saccades on average. We provide as supplementary material example videos of experimental trials as a function of mask type (central, peripheral mask, and no mask). These examples help to illustrate the strong forward motion momentum of the head, and the smaller movements of the eye happening during large head shifts and when the head is at a relative rest.

### Time-course of free-viewing tendencies

**Figure 9.**
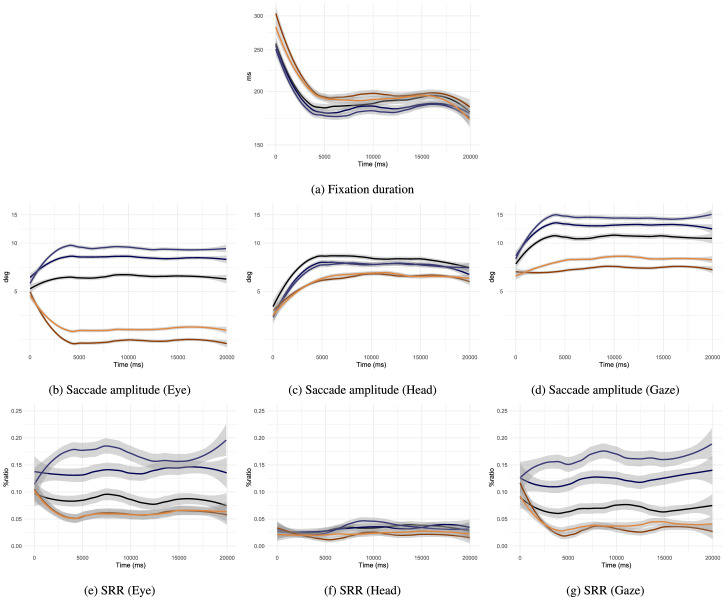
Mean and 95% CI of visuo-motor variables as a function of viewing time. Colour legend: 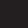 no-mask, 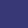 central 6°, 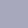 central 8°, 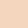 peripheral 6°, 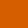 peripheral 4°. Fixation durations and saccade amplitudes are displayed on a log-scale.

Because local and global viewing cognitive processes influence saccade and fixation dynamics, it is important to acknowledge their time-varying characteristics when predicting or modelling gaze movements. In this subsection we plot a selection of variables reported on above as a function of viewing time (Figure 9). We removed from our analysis the last saccade and fixation occurring in trials; because the abrupt end of stimulus presentation could artificially lower the average fixation durations and saccade amplitudes sampled at the end of trials. Past research has demonstrated that gaze behavior can vary with time in regard with visuo-motor statistics (e.g., Unema et al., 2005; Tatler & Vincent, 2009) or attentional guidance (e.g., Rai et al., 2016; Theeuwes et al., 2000). Free-viewing in VR is no exception in relation to fixation duration and saccade amplitude in particular. In this study, we note that average fixation durations at the start of trials are the highest observed (Figure 9a). After trial onset the average duration promptly decreased and stabilized after 5 seconds of viewing time. Longer fixation durations can be evidence for difficulty to process the content of the field of view (van Diepen & d’Ydewalle, 2003; Nuthmann et al., 2010; Laubrock et al., 2013), possibly in this case to construct a first representation of the scene (van Diepen & d’Ydewalle, 2003; Castelhano & Henderson, 2007; Larson & Loschky, 2009). However, the fact that the no-mask condition also shows an increase in fixation duration at first allows us to rule out an effect related to visual processing complications related to gaze-contingent masking. As we discuss elsewhere in this article, we believe it to be related to our choice of visual task. After approximately 17 second average durations slightly decreased again until trial ends. This time-course pattern of fixation duration was repeated in presence of gaze-contingent masks.

Eye movement amplitude during saccade increased by 1° in the no-mask condition over 2.5 seconds and was fairly stable across trial duration past this point (Figure 9b). Participants may have produced smaller eye movements at the starts of a trial to build a gist of the scene appearing at first in the displays. Interestingly, even when experiencing vision loss, participants started by making eye movements similar to the control condition. The increase in average amplitudes observed during central-masking trial and the decrease during peripheral-masking trials was really effective after 2.5 seconds of viewing time; the impact of masking on the visual system may affect motor programming with a delay. It was observed that head movements are decreased at the start of visual search trials (David et al., 2020); this is also the case for free-viewing, in control and masking conditions alike (Figure 9c). An observer does not immediately start exploring an omnidirectional scene with large shifts of the field of view, preferring to slowly increase head use over 5 seconds. Beyond that 5-seconds mark, head movement amplitudes are somewhat stable until the end of the trial. The two preceding movements combine to produce the final gaze saccade. We notice that average saccade amplitudes started short, again strongly increasing as observers approached 5 seconds of viewing time (Figure 9d). This was less true for peripheral-masking trials where eye movements were significantly reduced, despite head movements the resulting gaze movement amplitude did not increase nearly as much as in the other conditions.

Time-course analysis of relative saccade direction measure (SRR) shows that the average rate of backward saccades was fairly stable across time without mask, as seen through eye, head and gaze movements (Figures 9e, 9f and 9g). We hypothesize that a refixation behavior was not particular to any time point, previous research showed that observation of local and global scenes features was intermixed after the first second of observation (Antes, 1974; Rai et al., 2016). The masking effect on backward saccade rates of eye and gaze movements reported in the previous section are particularly apparent after approximately 2 seconds of viewing time. On another hand, head relative directions vary less across time and masking conditions.

## General discussion

In this experiment, we simulated central and peripheral loss of vision with gaze-contingent masks on omnidirectional stimuli presented within a VR headset. Our main results reveal how observers navigate 360 scenes with their eyes and head, as well as how their behaviours evolved over time. We showed that the effects of transient loss of vision are particularly apparent through eye movements. In comparison, head movement amplitudes are decreased without significantly affecting absolute and relative head direction dynamics. We believe that two distinct behaviors appear in our results. The analysis of regions of interest happens through eye movements much more than head movements. In contrast, the exploration of omnidirectional scenes relies on gaze movements of big amplitude (compared with usual on-screen stimuli), enlisting the head more than the eyes. A time-course analysis showed strong differences with the literature that are discussed in this section.

It is important to repeat that head movements in this study were sampled from the headset's 3D tracking system. As such, head rotations were affected by rotations of the neck, but also of the rest of the body, and of the swivel chair in which participants were sitting. Our data do not allow separating the precise source of the head rotation.

### Impact of gaze-contingent masking in VR

We generally replicated the effects of gaze-contingent masking reported on screen: an increase in saccade amplitude without central vision and a decrease with peripheral masking (e.g., Foulsham et al., 2011a; Nuthmann, 2014; Cajar et al., 2016a; Geringswald et al., 2016); an increase in backward saccades when central vision is missing (Henderson et al., 1997), an effect that is correlated with the mask radius (David et al., 2019). Similar to on screen (David et al., 2019), average fixation durations were reduced in the presence of a central mask, and more severely so with a bigger mask. We hypothesize that fixations are shorter because of the lack of central information to process and a particular behavior where participants produced several return saccades interspersed when trying to analyze an object of interest (as described by Henderson et al., 1997). This is probably only observed because there is little top-down pressure to finely explore the scene. As expected, in the absence of peripheral information there was very little visual content to refixate, as a result the average number of backward saccades was decreased (David et al., 2019), and the average length of the combined gaze's forward segments (FSSL) increased. Without peripheral vision participants made longer fixations, as was observed on screen as well (David et al., 2019). Because less central information is actually left to be analyzed, and under the hypothesis that a uniformly grey peripheral field of view is increasing peripheral processing times, we conclude that the time increase reported is in relation to saccade planning made more difficult by the lack of peripheral vision. Overall, head movements amplitudes were significantly reduced during trials with masks. This observation possibly reflects the unease of wearing a VR headset.

### The role of head and eye movements

Most eye movements recorded in this study were below 15° of amplitude (87.9%), and the head was used to extend the normal field of fixation making the final average gaze movements at least twice as large as would be observed on screen (between 4° and 7° on average; see, e.g., Laubrock et al., 2013; Cajar et al., 2016b; Nuthmann & Malcolm, 2016). The combined gaze movement is not a simple combination of eye rotations with head motions, they can be directed in opposite directions (in case of backward saccades during a long head movement). By studying the distribution of absolute and relative head movement directions, as well as FSSL, we have showed that the head serves to explore the scene horizontally by panning the field of view in the same direction over what constitutes several combined gaze saccades. This was not seriously impacted by gaze-contingent masks. The head shifts the field of view in one direction horizontally, while the eyes make several compensatory movements to analyse the visual content appearing in the displays. The exploratory role of the head, with long sequence of movements in the same direction, may be overestimated in this study, due to the ease with which a participant could use the chair to rotate the environment. As was reported on screen (e.g., Foulsham et al., 2011a; David et al., 2019) and in VR (David et al., 2020), eye movements are strongly impacted by visual stimulation; they target predominantly visible areas of the field of view. Interestingly, with a peripheral mask, we noticed that the combined head and eye movement often resulted in saccades targeting the masked part of the field of view (at the time of saccade planning). We reported this finding previously in the context of visual search in VR (David et al., 2020). We believe that eye movements, being bound by visual stimulation, and head movements bringing gaze into masked areas of the field of view are clues that eye and head movement programming is dissociated in regard to their dependency on visual stimuli and exploratory behaviors, respectively (Anderson et al., 2020; Bischof et al., 2020). We hypothesize that head movements can be more easily shaped by task-related goals, even in the presence of masks. It emerged that eye movements are complex and will explore or analyze the content of the field of view, while the head moves in simpler ways (in long horizontal spanning movements), and with the help of the rest of the body, works to uncover the whole 360° scene.

### Time-course tendencies of VR free viewing

Taken together, those results show a strong stability for all measures beyond a few seconds past trial onset. Trials started with long fixations and short saccades, long fixations served to thoroughly exploit central as well as peripheral contents (Ehinger et al., 2018). Exploration of the omnidirectional scene was really set in motion 5 seconds after trial onset as average fixation durations had become shorter and average saccade amplitudes substantially longer. This warming-up delay needed to explore the initial field of view before making significant head movements has been reported in visual search in VR as well (David et al., 2020), and was interpreted as a second search initiation time of head stability to grasp the content of the displays before moving to the rest of the scene. It may be needed by an observer to find their bearings at first in 360 conditions. Starting trials with long fixations and short saccades is in contradiction with the literature on screen (Antes, 1974; Unema et al., 2005; Eisenberg & Zacks, 2016; Ito et al., 2017), reporting the inverse effect: shorter fixations and longer saccades at scene onset to explore the scene with more fixations possibly covering more of the scene's content quickly. One key difference between these studies and ours is the choice of visual task. Antes (1974) asked of viewers to judge art pictures according to their preferences; Unema et al. (2005) let participants view scenes before asking questions about their content, and Eisenberg and Zacks (2016) implemented a scene video segmentation task (in their second experiment). The closest experiment to free-viewing would be one by Ito et al. (2017), the authors trained monkeys on a visual task with an incentive to keep gaze within the screen's boundaries. Four of the five mentioned studies have in common to have constrained viewing by adding a task, whereas we explicitly told participants that we expected them to freely explore without any afterthought. To strengthen our point, we provide a time-course analysis of the data from an on-screen free-viewing (David et al., 2019) in the Appendix (section A.2). We do not believe the time-course effects observed in the present study to be the result of moving from on-screen to VR because it was not observed in visual search in VR (David et al., 2020). We hypothesize that the absence of task and the low top-down requirement that follows are the cause of the differences observed with experiments implementing constrained viewing.

### Viewing tendencies

In this study, we have described tendencies related to head and eye behaviors during fixations and saccades. A better understanding of how the head moves is no doubt critical to compression and streaming methods based on predicting what part of the omnidirectional stimulus to serve in the next second (Li et al., 2019). As expected, we did not show sizeable variations in absolute angle measures as a function of masking conditions. As a result, the horizontal bias in VR only varied with mask types by its amplitude. We have shown that the head generally spans scenes with movements long in duration and amplitudes; consequently, future head movements during free-viewing can be predicted with a time-dependent model (Nguyen et al., 2018; Rondón et al., 2020). Because in the displays’ frame of reference the eyes do not move significantly far from the centre (Sitzmann et al., 2017), a big part of the peripheral content presented in the headset could be decreased in quality as a function of eccentricity, foveated rendering would allow compressing visual information further (Weier et al., 2017). In addition to the tendencies discussed so far we share general and viewport-based biases in section A.3 of the Appendix. We believe that our results particular to head-free exploring of omnidirectional environments can be leveraged to create powerful gaze models or adapt existing ones to virtual reality conditions.

Finally, it must be reiterated that our results originate from the free-viewing of nonstereoscopic omnidirectional stimuli. Furthermore, the scenes viewed by the subject were in most cases 360 photographs of large open spaces. This may have had an effect on visuo-motor behaviours by eliciting more exploratory behaviours to the detriment of the analysis of regions of interest. We must stress that some effects observed in this study may not replicate with more directed tasks (e.g., visual search); as such, a model of eye movements based on the present findings may only be suitable for free-viewing of natural scenes. Additionally, the experimental setup (material and the nature of the stimuli) and its limitations (e.g., frame rate, graphical display resolution, sitting on a swivel chair) may have produced, in part, unnatural behaviors.

## Conclusion

With this experiment we set out to answer what fixation and saccade tendencies could be measured in VR? The implicit corollary to this question was: do these tendencies differ from the ones observed on screen? Our results replicate overall on-screen findings about saccade absolute and relative directions tendencies. One key difference is the observation that participants started trials with long fixations and short saccades. We justified this difference to be related to our choice of task rather than the VR settings, that is because we showed the same effect in a previous similar on-screen protocol. The addition of head rotations sampling taught us that the head generally contributes to scene viewing by extending the field of fixation with horizontal and forward movements to scan the scene. In contrast eye movements seemed to account for finer exploration and analysis of the displays’ content. Our main finding related to our implementation of gaze-contingent masking is that eye movement programming is strongly impacted by visual stimulation, whereas head movements can serve a task goal of exploration despite masking. We hope that this study will encourage the replication of gaze-contingent experiments in VR, to study the role of eye and head movements, as well as the role of central and peripheral vision in more naturalistic conditions without the need for mobile eye-tracking or complex apparatus.

## Supplementary Material

Supplement 1

Supplement 2

Supplement 3

## Figures and Tables

**Figure A.1.**
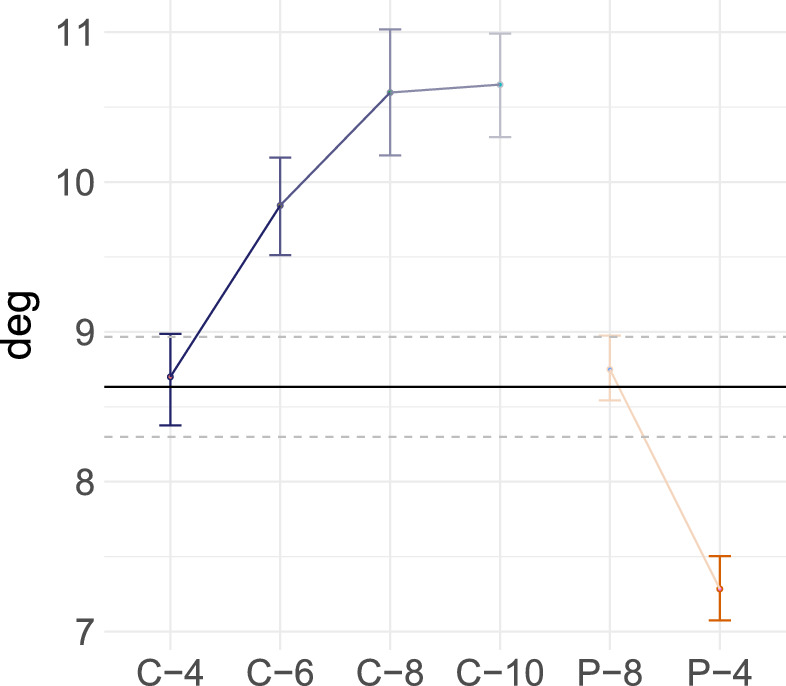
Average amplitudes and confidence intervals (95%) of gaze saccades as a function of mask types and radii. “C-4” refers to a central mask of 4° of radius, “P-6” to a peripheral mask of 6°.

**Figure A.2.**
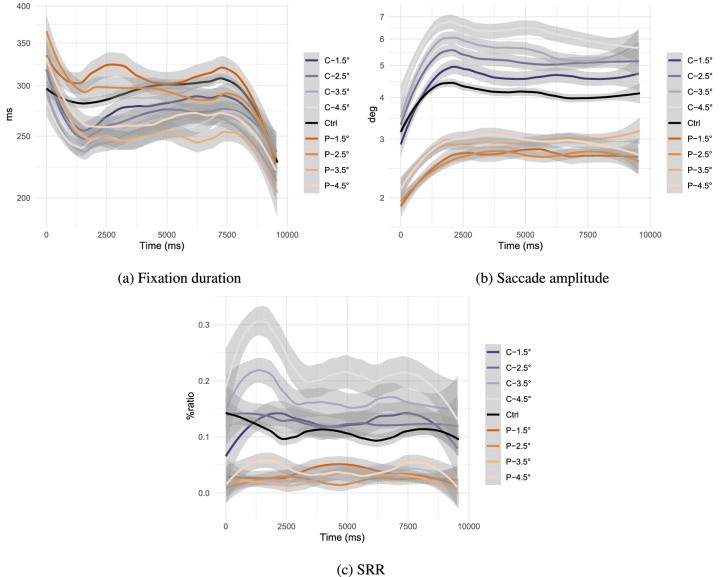
Time-course average of oculo-motor variables gathered on-screen in David et al. (2019). We report time-dependent means (coloured lines) and 95% CI (grey ribbons) for control (Ctrl), central-masking (C-*), and peripheral-masking (P-*) conditions. The numbers after C- and P- refer to the gaze-contingent mask's radius. Fixation durations and saccade amplitudes were log-transformed to better estimate distribution means. Fixation durations and saccade amplitudes are displayed on a log-scale.

**Figure A.3.**
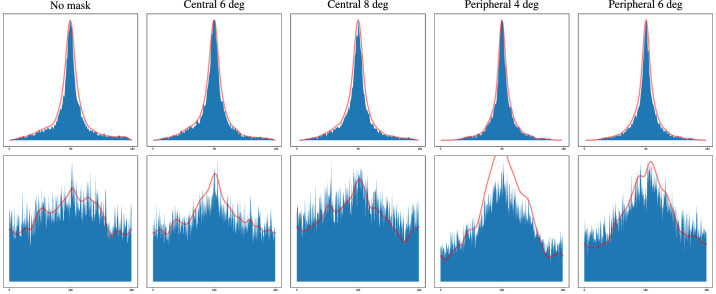
Fixation position count (blue) by latitude (top, 0${}^{\circ }$ to 180${}^{\circ }$) and longitude (bottom, -180${}^{\circ }$ to 180${}^{\circ }$). PDF curves (red) are fitted with a Gaussian kernel for latitudes and a von Mises kernel for longitudes. The first second of the dataset was removed to decrease an effect of the starting position.

**Figure A.4.**
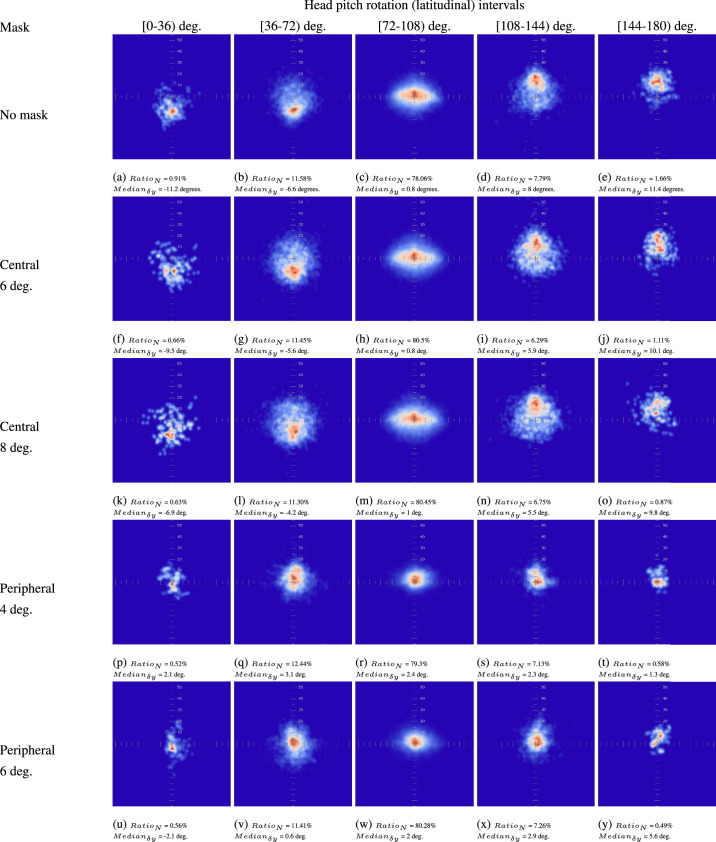
Density distribution estimation (by Gaussian kernel) of fixation positions in the viewport as a function of the headset's latitudinal rotation (*pitch*). Below each subfigure is reported the number of fixations sampled in the latitudinal range divided by the total number of fixations ($Ratio_{N}$). $Median_{\delta y}$ informs about the median distance of the fixation positions on the vertical axis to the center of the viewport.

## Appendix

### A.1 A1 Pre-study

To validate the experimental protocol and choose mask radii we set up a prestudy. We wanted to select central masks that were not so small that participants would behave as in the no-mask condition and not so big that the effects would plateau. As for peripheral loss, we wished for masks that were not so small as to render blind but not so big that participants would behave without any hindrance. For these reasons we recruited five naïve subjects; they observed the 28 omnidirectional static scenes during 30 seconds, experimenting with six gaze-contingent masks: four central (4°, 6°, 8°, 10°) and two peripheral (4°, 8°). More central mask sizes were tested because this masking condition is critical in regard to eye tracking precision and overall system latency. We made our decision on the basis of average (combined gaze) saccade amplitudes, which is strongly and stereotypically impacted by artificial vision losses (e.g., Nuthmann, 2014; David et al., 2019; Figure A.1). We rejected the smallest and biggest central mask because the first made subjects behave like in the no-mask condition and the second seemed to have reached a plateau in its impact. We assumed the effect to vary as a function of peripheral mask size, then substituted the larger peripheral mask (8°) with one of 6° to observe an evolution in the behavior as a function of mask size without one of the mask resulting in behaviours similar to the control condition. Four masks were ultimately selected in order to keep a suitable statistical power.

### A.2 A2 Time-course analyses of on-screen data from David et al. (2019)

The present experiment replicates a previous study implementing transient loss of vision on screen with central or peripheral masking with varying mask radii (David et al. 2019). Participants had 10 seconds to freely view pictures of natural scenes on screen. A gaze-contingent mask would remove part of their central or peripheral field of view in real time. Because we used an eye tracker with a chin rest, only eye movements were measured. To compare time variations of variables reported in this VR study to on-screen data, we analyzed data from David et al. (2019), plotting variable means as a function of viewing time (Figure A.2). We removed data related to the last fixation in trials.

We see an increase in the average fixation durations at trial onset, particularly present with gaze-contingent masking, the effect on no-mask trials is not as strong. We also observe shorter saccade amplitudes at scene onset compared with the rest of the trials. The average saccade amplitudes increase to reach a plateau after 2.5 seconds of viewing time, for all masking and the control conditions. Measuring SRR as a function of time, we see a temporary increase in backward saccade rates approximately a second after scene onset when considering the largest central masks (3.5° and 4.5° of radius). Indeed, free-viewing seems to affect the fixation and saccade dynamics at trial onset in the same manner on screen as in VR.

### A.3 A3 General and viewport-based fixational biases

Central fixation bias in scene viewing has been reported on extensively (e.g., Tatler, 2007; Tseng et al., 2009; Bindemann, 2010), it is so prevalent that it is used as a baseline for saliency model benchmarking (Bylinskii et al. 2015). Center biases are observed in omnidirectional contents as well, in particular we observe an equator bias (latitudinal centre bias): most fixations are located near the equator Figure A.3. The addition of gaze-contingent masks does not seem to modify this bias significantly.

The decreased longitudinal center bias in Figure A.3 indicates that the longitudinal center bias observed in the literature (David et al., 2018; Xu et al., 2018) is probably due to photographic and cinematographic tendencies to center an object of interest in the scene and to an experimental bias related to starting trials at a longitudinal rotation shared between observers. Studies implementing different starting longitudinal rotations (Sitzmann et al., 2017; David et al., 2018) suggest that participants converge to similar points of interests after a few seconds of observations. With the addition of gaze-contingent masks this bias seems to grow stronger, in particular with peripheral masks; the fact that the average saccade amplitude is decreased when the peripheral field of view is masked means that participants explored less away from the starting position. Artificial loss of vision did not particularly affect the distribution of fixation longitudinally.

In Figure A.4, we present the spatial distribution of fixations as a function of head pitch (rotation on the longitudinal axis, from south to north). We observe that the eye and head movements are correlated: the eye positions are biased downward in the viewport when the head is rotated downward, and biased upward in the viewport when the head is rotated upward.

In cases of simulated vision loss, eye positions seem to be somewhat restrained in the viewport; they do not follow vertical head rotations as much. This effect is particularly salient with peripheral masks where we notice that eye positions are more centred in the viewport, with less dispersion on the horizontal axis as well.
